# Effectiveness of high frequency ultrasound on pressure ulcer: A systematic review protocol of randomized controlled trial

**DOI:** 10.1097/MD.0000000000017111

**Published:** 2019-09-13

**Authors:** Xiang-qin Gao, Xiao-mei Xue, Jian-kang Zhang, Fen Yan, Qiu-xia Mu

**Affiliations:** Department of Ultrasound, Yulin No. 2 Hospital, Yulin, China.

**Keywords:** effectiveness, high frequency ultrasound, pressure ulcer, randomized controlled trial

## Abstract

**Background:**

This study aims to systematically assess the effectiveness of high frequency ultrasound (HFUS) on pressure ulcer (PU).

**Methods:**

In this study, PubMed, EMBASE, Cochrane Library, Web of Science, Chinese Biomedical Literature Database, and China National Knowledge Infrastructure will be searched from inception to the present without any language limitations. The primary outcomes include change in ulcer area, and time complete healing. The secondary outcomes consist of proportion of ulcers healed within trial period, quality of life, pain intensity, and adverse events. Cochrane risk of bias tool will be used to assess methodological quality. RevMan 5.3 software (London, UK) will be used to analyze the data.

**Results:**

This study will analyze change in ulcer area, time complete healing, proportion of ulcers healed within study period, quality of life, pain intensity, and adverse events on HFUS in patients with PU.

**Conclusion:**

This study will provide most recent evidence for the effectiveness and safety of HFUS for patients with PU.

**PROSPERO registration number:**

PROSPERO CRD42019138177.

## 1 Introduction

Pressure ulcer (PU) is area of localized injury to the skin and its underlying tissue.^[1,2]^ It often occurs in the elderly who lose their ability to reposition themselves to avoid much pressure on bony prominences.^[3–5]^ The most common anatomical locations for PU to occur consist of sacrum, hips, heels, and elbows.^[6–8]^ It has been reported that its prevalence rate varies from 8.8% to 53.2%, and its incidence rate ranges from 7% to 71.6%.^[9–12]^ Additionally, this order has also been associated with an increased incidence of infection,^[13,14]^ and can greatly decrease quality of life in patients with PU.^[15,16]^

Previous studies have reported that high frequency ultrasound (HFUS) is widely used for the management of PU.^[17–25]^ Unfortunately, there is no systematic review to investigate the effectiveness and safety of HFUS for the treatment of PU. Therefore, this study will systematically assess the effectiveness and safety of HFUS therapy for the management of patients with PU.

## 2 Methods

### 2.1 Criteria for considering studies for this study

#### 2.1.1 Types of studies

Randomized controlled trials (RCTs) that assess the effectiveness and safety of HFUS for patients with PU will be included irrespective of language. However, we will exclude studies that do not belong to the RCTs.

#### 2.1.2 Types of participants

Study involving patients with existing PU of any grades will be considered for inclusion regardless of race, gender, etc.

#### 2.1.3 Types of interventions

All patients in the treatment group receive HFUS intervention.

All patients in the control group receive any treatments, except any forms of HFUS.

#### 2.1.4 Types of outcome measurements

The primary outcomes include change in ulcer area (surface area change since baseline), and time complete healing (complete healing was defined as intact skin).

The secondary outcomes consist of proportion of ulcers healed within trial period, quality of life (measured by any related scales), pain intensity (measured by visual analogue scale or any others), and adverse events.

### 2.2 Search methods for identification of studies

The following electronic databases will be searched: PubMed, EMBASE, Cochrane Library, Web of Science, Chinese Biomedical Literature Database, and China National Knowledge Infrastructure. All of them will be searched from inception to the present without language restrictions. The provisional search strategy to be used in PubMed can be found in Table 1. Similar search strategy will be adapted to other electronic databases.

**Table 1:**
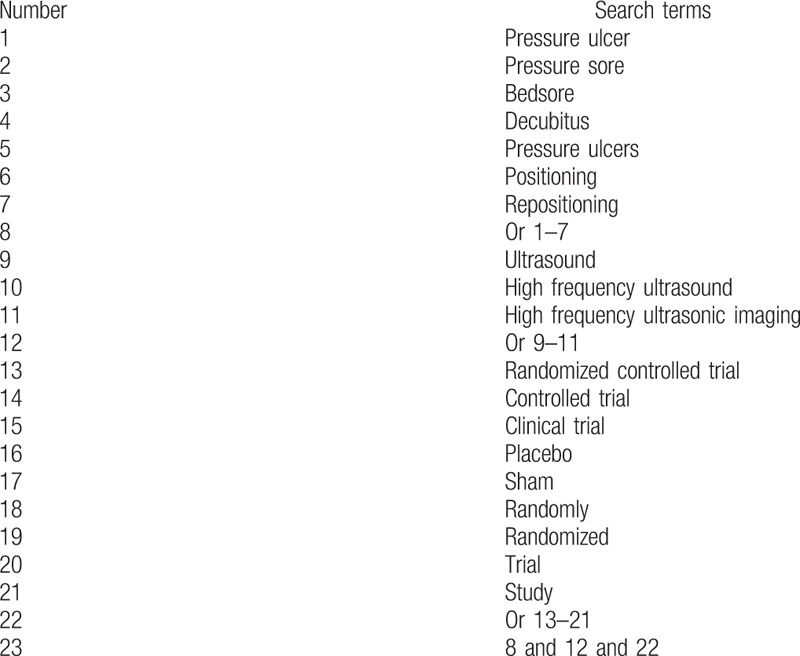
Search strategy applied in PubMed database.

In addition, reference list of relevant reviews and dissertations will also be checked to identify any potential eligible studies.

### 2.3 Data collection and analysis

#### 2.3.1 Selection of studies

**Figure 1:**
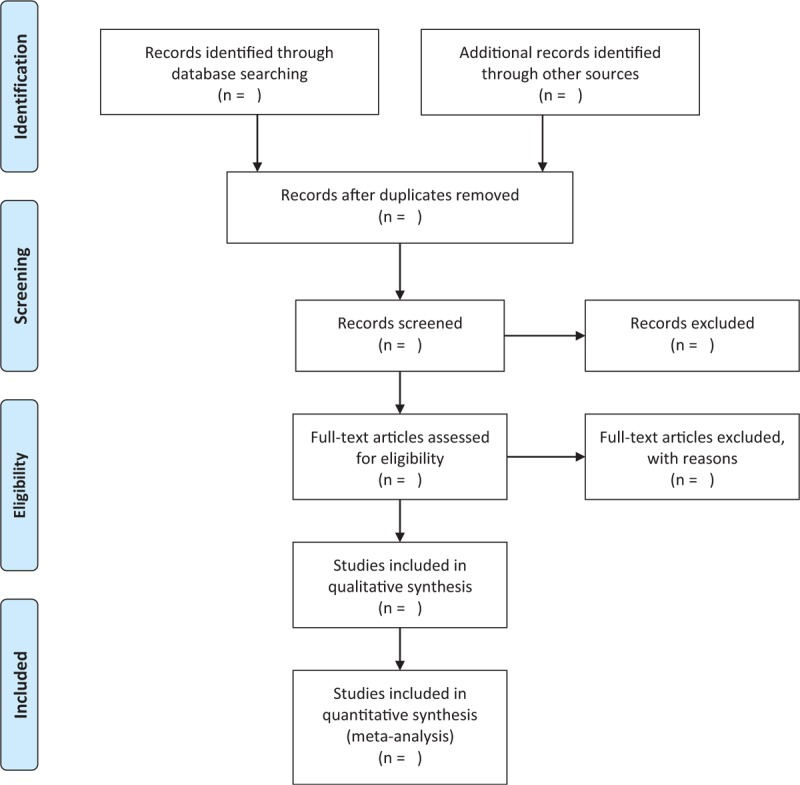
Flowchart of study selection.

Two authors will independently evaluate the titles and abstracts of each potential record identified by the eligibility criteria. Full texts of remaining records will be further assessed by the same 2 authors against all the inclusion criteria. Any disagreements will be resolved by consensus with a third experienced author when necessary. The process of study selection will be showed in the flowchart in Figure 1.

#### 2.3.2 Data extraction and management

Two authors will independently extract data using predefined data extraction form. Divergences regarding data extraction between 2 authors will be solved by a third experienced author through discussion. The following information will be extracted from each eligible study.

Study characteristics: title, author, year of publication, location, etc.

Patient characteristics: diagnostic criteria, gender, age, etc.

Study methods: sample size, randomization methods, allocation concealment, etc.

Details of treatment and comparator: dosage, frequency, duration, etc.

Outcome measurements: primary and secondary outcomes, safety, etc.

#### 2.3.3 Missing data management

When there is evidence of data missing or insufficient, we will attempt to contact primary authors using email. If we cannot achieve those data, we will analyze available data, and their impacts will be discussed.

#### 2.3.4 Risk of bias assessment

Two authors will independently evaluate included studies using Cochrane Risk of Bias Tool. This tool addresses 7 aspects, and each aspect is further classified as 3 levels of low, unclear, and high risk of bias. A third experienced author will be asked to help resolve disagreements between 2 authors through discussion.

#### 2.3.5 Data synthesis and analysis

We will use RevMan 5.3 software (London, UK) to synthesize and analyze the data. For time to event data, it will be expressed as hazard ratios. For continuous data, it will be expressed as mean difference or standardized mean difference with 95% confidence intervals. For dichotomous data, it will be exported as risk ratio with 95% confidence intervals.

In this study, we will apply *I*^2^ test to check heterogeneity. The guide of *I*^2^ value is interrelated as follows: 0% to 50%: may represent reasonable heterogeneity; and 51% to 100%: may represent significant heterogeneity. When there is reasonable heterogeneity, a fixed-effects model will be used for data pooling; and meta-analysis will be conducted. When there is significant heterogeneity, a random-effects model will be used and subgroup analysis will be performed. When there is still substantial heterogeneity after subgroup analysis, data will not be pooled, and meta-analysis will not be performed. On the other hand, only narrative summary will be described for outcome results.

#### 2.3.6 Additional analysis

We will perform subgroup analysis in accordance with the different treatments, controls, and outcome measurements. In addition, we will also carry out sensitivity analysis to check the robustness of outcome results by removing low-quality studies.

#### 2.3.7 Publication bias

Publication bias will be checked using funnel plot^[26]^ and Egger regression^[27]^ if sufficient studies are entered in this study.

## 3 Discussion

PU is a very common disorder in the elderly. Previous studies have reported that HFUS is effectively used for the treatment of patients with PU. However, up to the present, there has no relevant systematic review been reported. This study aims to objectively provide reliable evidence of HFUS for the treatment of patients with PU based on evidence-based medicine, and whether it can effectively manage PU. Its results will provide rigorous evidence and will inform our understanding of HFUS for patients with PU across all published RCTs.

## Author contributions

**Conceptualization:** Xiao-mei Xue, Jian-kang Zhang, Fen Yan, Qiu-xia Mu.

**Data curation:** Xiang-qin Gao, Fen Yan, Qiu-xia Mu.

**Formal analysis:** Xiang-qin Gao, Xiao-mei Xue, Jian-kang Zhang, Fen Yan.

**Investigation:** Fen Yan, Qiu-xia Mu.

**Methodology:** Xiang-qin Gao, Jian-kang Zhang.

**Project administration:** Qiu-xia Mu.

**Resources:** Xiang-qin Gao, Xiao-mei Xue, Jian-kang Zhang, Fen Yan.

**Software:** Xiang-qin Gao, Xiao-mei Xue, Jian-kang Zhang, Fen Yan.

**Supervision:** Qiu-xia Mu.

**Validation:** Xiang-qin Gao, Qiu-xia Mu.

**Visualization:** Xiang-qin Gao, Xiao-mei Xue, Jian-kang Zhang, Fen Yan, Qiu-xia Mu.

**Writing – original draft:** Xiang-qin Gao, Xiao-mei Xue, Jian-kang Zhang, Fen Yan, Qiu-xia Mu.

**Writing – review & editing:** Xiang-qin Gao, Xiao-mei Xue, Jian-kang Zhang, Fen Yan, Qiu-xia Mu.
