# Effectiveness of high frequency ultrasound on pressure ulcer: A systematic review protocol of randomized controlled trial: Erratum

**DOI:** 10.1097/MD.0000000000017485

**Published:** 2019-10-04

In the article, “Effectiveness of high frequency ultrasound on pressure ulcer: A systematic review protocol of randomized controlled trial”,^[1]^ which appears in Volume 98, Issue 37 of *Medicine*, Fei Yan's name appeared incorrectly as Fen Yan.

## References

[1] Gao XQ Xue XM Zhang JK Effectiveness of high frequency ultrasound on pressure ulcer: A systematic review protocol of randomized controlled trial. *Medicine.* 2019 98:e17111. 31517845 10.1097/MD.0000000000017111 PMC6750305

